# *Aedes albopictus* in a recently invaded area in Spain: effects of trap type, locality, and season on mosquito captures

**DOI:** 10.1038/s41598-024-52040-4

**Published:** 2024-01-25

**Authors:** Mario Garrido, Jesús Veiga, Marta Garrigós, Manuel Morales-Yuste, Jesús Recuero-Gil, Josué Martínez-de la Puente

**Affiliations:** 1 Department of Parasitology, University of Granada (UGR), Granada, Spain; 2 Bioparc Fuengirola, Fuengirola, Málaga Spain; 3 Doñana Biological Station (EBD, CSIC), Sevilla, Spain; 4 CIBER de Epidemiología y Salud Pública (CIBERESP), Madrid, Spain

**Keywords:** Entomology, Infectious diseases

## Abstract

Mosquitoes are primary vectors of pathogens impacting humans, wildlife, and livestock. Among them, the Asian tiger mosquito, *Aedes albopictus*, stands out as an invasive species with a global distribution, having established populations on every continent except Antarctica. Recent findings incriminate *Ae. albopictus* in the local transmission of several pathogens causing human diseases, including dengue, chikungunya, and Zika viruses and worm parasites as *Dirofilaria*. In Spain, the establishment of *Ae. albopictus* occurred in 2004 and it rapidly expanded, currently reaching southern provinces and creating novel epidemiological scenarios in recently invaded areas. In this study, we conducted captures of *Ae. albopictus* from May to November 2022 in two provinces, Granada and Malaga, situated near the current edge of the species' expanding range in Spain. The objective was to identify the primary factors influencing their captures in these regions. Mosquitoes were captured using BG-Sentinel traps baited with CO_2_ and BG-Lure, and miniature CDC-UV traps in five different localities. Our findings underscore the influence of both extrinsic factors, such as locality, and intrinsic factors, including mosquito sex, on the abundance of captured *Ae. albopictus*. A higher abundance of *Ae. albopictus* was observed in the Malaga province compared to localities in the Granada province. Furthermore, similar numbers of *Ae. albopictus* mosquitoes were captured in more urbanized areas of Granada, while the lowest counts were recorded in the less urbanized area. These results were compared to captures of another common species in the area, specifically *Culex pipiens*. Overall, these results represent the first monitoring of invasive *Ae. albopictus* in the area and are discussed in the light of the potential importance of the species as a nuisance for humans and vectors of pathogens of public health relevance.

## Introduction

Emerging infectious diseases (EIDs) have spread globally in the last decades, drove by human-induced environmental changes^1,2^. Most EIDs are transmitted by insects, with mosquitoes serving as the primary vectors of pathogens that impact both humans and other animals^3–5^. Mosquito-borne pathogens include relevant parasites like *Plasmodium* and filarial worms, and viruses responsible for diseases such as yellow fever, dengue, or West Nile fever, producing human fatalities worldwide^3,6^. Yet, only about 10% of the known species of mosquitoes are vectors of pathogens affecting human populations^7^. Among them, mosquitoes of the *Aedes* genus represent one of the most relevant vectors, including the noteworthy invasive yellow fever mosquito *Aedes aegypti* and the Asian tiger mosquito *Aedes albopictus*.

*Aedes albopictus* is an invasive species broadly distributed in most of the continents around the globe^8^, cataloged as one of the top 100 most dangerous invasive species^9,10^ due to its capacity to adapt and colonize new areas and to spread zoonotic arboviruses^8,11^. In Europe, *Ae. albopictus* was first introduced in Albania in the 70`s but, nowadays, it has established populations in more than 15 European countries, including all those of the Mediterranean basin, and some Eastern (e.g., Bulgaria, Romania) and Central European (e.g., Germany, Switzerland, or Belgium) countries^12^. In the European invaded areas, this species is recognized as an important nuisance for humans due to their bites. In addition, *Ae. albopictus* is a significant public health concern due to its involvement in the local transmission of both native and imported pathogens. This includes *Dirofilaria* parasites in Italy^13,14^ and viruses such as Zika virus, dengue virus, and chikungunya virus in France, Italy and Spain^15–20^. In Spain, *Ae. albopictus* was first identified in 2004 in the province of Barcelona^21^, but it has rapidly invaded other areas of the country likely favored by factors including the passive dispersal in private vehicles^22^. Nowadays, the species has reached different provinces in Spain including those of the southern Andalusian region^23,24^. In the provinces of Granada and Malaga, *Ae. albopictus* was first reported in 2014^23^. Given the public health significance of this species and the lack of information regarding its dynamics in these recently invaded areas, it is necessary to conduct monitoring studies to identify the primary factors affecting the local abundance of *Ae. albopictus* in southern Spain. This is especially relevant due to the frequent occurrence of imported cases of both dengue and chikungunya in the area^24^. For instance, between 2008 and 2020, Andalusia reported a total of 109 imported cases of dengue, with 35 cases recorded in 2019. Notably, the metropolitan area of Granada and the coastal area of Malaga provinces exhibited the highest number of cases^24^. Yet, at least for the case of dengue, there is a medium/low risk of local transmission in these areas^25^.

**Figure 1:**
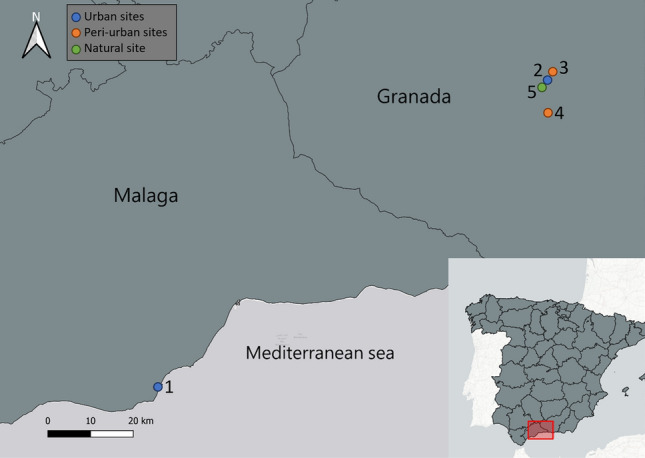
Mosquito sampling localities in the provinces of Granada (upper-right points) and Malaga (lower-left point), Spain, used during the study period. Two sampling points were designated at each locality for each session. Sampling localities: (1) Fuengirola (M.), (2) Fuentenueva (Gr.), (3) Cartuja (Gr.), (4) Gójar (Gr.), and (5) La Vega (Gr.). M. = Málaga; Gr. = Granada.

In this respect, we studied the influence of the sampling procedure employed (i.e., trap type) on mosquito captures by comparing results for both the invasive *Ae. albopictus* with those of the common house mosquito *Culex pipiens*. Secondly, representing our main goal, we evaluated the spatial and temporal variation of the captures of this invasive species and compared it with those of the *Cx. pipiens*. To achieve these goals, mosquitoes were sampled over ten trapping sessions distributed regularly from May to November 2022, using two different trap types at five different localities with different environmental characteristics (Fig. 1).

## Results

Overall, 1440 mosquitoes were captured in this study. The most common trapped species was *Culex pipiens* (n = 974; 763 females, 210 males, and 1 undetermined; Table 1) followed by *Culiseta longiareolata* (n = 225; 104 females, 120 males, and 1 undetermined), *Aedes albopictus* (n = 211; 139 females, 70 males, and 2 undetermined; Table 1), *Culiseta* spp. (n = 6; 4 females, 2 males), and *Anopheles atroparvus* (n = 3; all females). In addition, 21 mosquitoes were not identified due to the loss of morphological characters. Mosquitoes with undetermined sex were not included in subsequent analyses.

**Table 1:** Total number of *Aedes albopictus* and *Culex pipiens* captured at each sampling locality, categorized by trap type and sex.

	*Aedes albopictus*		*Culex pipiens*	
Sampling locality	BG-Sentinel	CDC-UV	BG-Sentinel	CDC-UV
Fuengirola (M.), urban site	73 (30♀, 43♂)	7 (3♀, 4♂)	202 (152♀, 50♂)	53 (17♀, 36♂)
Fuentenueva (Gr.), urban site	19 (16♀, 3♂)	3 (1♀, 2♂)	110 (101♀, 9♂)	18 (9♀, 9♂)
Cartuja (Gr.), peri-urban site	58 (48♀, 10♂)	0 (0♀, 0♂)	93 (91♀, 2♂)	9 (8♀, 1♂)
Gójar (Gr.), peri-urban site	45 (38♀, 7♂)	0 (0♀, 0♂)	119 (87♀, 32♂)	59 (17♀, 42♂)
La Vega (Gr.), natural site	4 (3♀, 1♂)	0 (0♀, 0♂)	285 (271♀, 14♂)	25 (10♀, 15♂)
*Subtotal by trap*	199 (135♀, 64♂)	10 (4♀, 6♂)	809 (702♀, 107♂)	164 (61♀, 103♂)
*Total*	209* (139♀, 70♂)		973* (763♀, 210♂)	

The number of females (♀) and males (♂) captured are shown in parentheses. M. = Málaga, Gr. = Granada, CDC-UV = Blacklight (UV)-CDC Miniature traps (Centers for Disease Control and Prevention, Atlanta, USA); BG-Sentinel = Biogents (BG)-Sentinel-2 traps (Biogents, Regensburg, Germany).

*Mosquitoes with undetermined sex, including two *Ae. albopictus* and one *Cx. pipiens*, have been excluded from this table.

### Comparison of the capture efficiency using BG-Sentinel and CDC-UV traps

Overall, out of 100 capture attempts using the two trap types in the 5 localities throughout the capture season (see methods), BG-Sentinel traps captured *Ae. albopictus* in 46% of occasions, while in CDC-UV traps this percentage dropped to 5%. This resulted in significant differences between trap types (Pearson's Chi-squared test with Yates' continuity correction: χ^2^ = 42.11, *p* < 0.001). Likewise, BG-Sentinel traps captured *Cx. pipiens* 93% of the occasions, whereas CDC-UV traps achieved a rate of 52% (χ^2^ = 40.13, *p* < 0.001). Differences in trap performance persist when analyzing the sexes of each species separately (all *p* < 0.05), except for *Cx. pipiens* males, which were trapped in similar percentages in both trap types (χ^2^ = 0.76, *p*-value = 0.38).

Abundance data show the same trends. The number of *Ae. albopictus* and *Cx. pipiens* mosquitoes captured using BG traps (*Ae. albopictus*: n = 199, x̄ ± SE = 1.99 ± 0.41; *Cx. pipiens*: n = 809, x̄ ± SE = 8.09 ± 0.79; Table 1) was higher than those using CDCs (*Ae. albopictus*: n = 10; x̄ ± SE = 0.10 ± 0.06; *Cx. pipiens*: n = 164, 1.64 ± 0.33 for CDC; Table 1). These differences reached significance for both *Ae. albopictus* (Mann–Whitney U = 7091.5, *p* < 0.001) and *Cx. pipiens* (Mann–Whitney U = 8569, *p* < 0.001). Once more, analyses segregated by sex and species reveal differences in the abundance of captures between trap types (all *p* < 0.05), except for male *Cx. pipiens* captures (Mann–Whitney U = 5385.5, *p* = 0.28).

### Effects of sex, locality, and seasonality on mosquito captures

**Figure 2:**
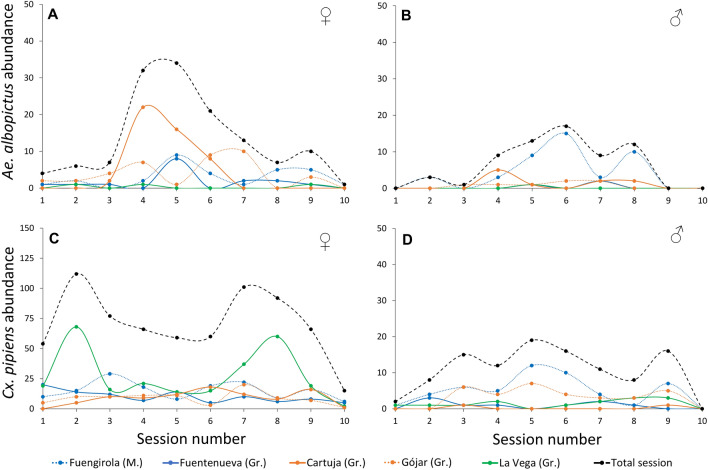
Population dynamics, estimated as the total number (abundance) of *Aedes albopictus* females (**A**) and males (**B**) and *Culex pipiens* females (**C**) and males (**D**) trapped using BG-Biogents (BG)-Sentinel-2 traps supplemented with dry ice as a source of CO_2_ and BG-Lure.

The summary of mosquito captures is found in Table 1. Results of GLM models are shown in Table 2. For the case of *Ae. albopictus*, locality (F_4,94_ = 5.52, *p* < 0.001; η^2^ = 0.18) and sex (F_1,98_ = 7.10; *p* < 0.01; η^2^ = 0.06) significantly affected the number of mosquitoes captured (*R*^2^ = 0.24). As expected, we captured a higher number of females than males. Post-hoc tests revealed differences in the number of *Ae. albopictus* captured between some localities (Fig. 2), with greater captures in the urban location at Fuengirola (n = 73) than in both Fuentenueva urban site (n = 19) (t_94_ = − 3.06, *p* = 0.02) and La Vega natural site (n = 4) (t_94_ = 4.38, *p* < 0.001). In addition, captures from La Vega, the locality with the lowest number of *Ae. albopictus* captured, differed from those of the periurban site of Gójar (n = 45) (t_94_ = − 3.01, *p* = 0.03). No differences were found between the periurban site of Cartuja (n = 58) and any other locality.

**Table 2:** Best fitted Gaussian-GLMs for the log + 1 transformed data of the number of *Aedes albopictus* and *Culex pipiens* mosquitoes trapped using BG-Sentinel traps.

Independent vbles	df	Deviance	Resid. Df	Resid. Dev	F value	*p* value	η^2^
*Aedes albopictus* (*R*^*2*^ = 0.24)							
Location	4	12.2704	94	52.268	5.5169	< 0.001***	0.18
Sex	1	3.9496	98	64.538	7.1031	< 0.001**	0.06
*Culex pipiens* (*R*^*2*^ = 0.67*)*							
Location	4	13.107	95	111.327	7.2777	< 0.001***	0.11
Sex	1	64.653	94	46.674	143.5923	< 0.001***	0.52
Location:Sex	4	6.151	90	40.523	3.4156	0.012*	0.05

Each model is reported with *R*^*2*^, and the significance (*p* value) and variance explained (η^2^: eta squared) by each of the independent variable in the models.

The final model (*R*^2^ = 0.67) for *Cx. pipiens* retained the factors sex (F_1,94_ = 143.6, *p* < 0.001; η^2^ = 0.52), locality (F_4,95_ = 7.28; *p* < 0.001; η^2^ = 0.11), and their interaction (F_4,90_ = 3.42; *p* = 0.012; η^2^ = 0.05). Significant differences in male captures (Fig. 2) were found in post-hoc analyses. These differences were observed between (1) the peri-urban sites of Gójar (n = 32) and Cartuja (n = 2) (t_90_ = 3.47, *p* < 0.001), (2) the urban site of Fuengirola (n = 50) and the peri-urban site of Cartuja (t_90_ = 4.67, *p* < 0.001), and (3) the urban sites of Fuengirola and Fuentenueva (n = 9) (t_90_ = − 3.375, *p* < 0.01). The number of male *Cx. pipiens* from La Vega natural site (n = 14) did not significantly differ from any other locality. For *Cx. pipiens* females, significant differences in capture numbers were found between some localities (Fig. 2). In particular, La Vega (n = 271), the locality with the highest mosquito captures of this species, differed significantly from both the periurban sites of Gójar (n = 87) (t_90_ = 3.14, *p* = 0.02) and Cartuja (n = 91) (t_90_ = 3.40, *p* < 0.01). No significant differences were found between the urban sites of Fuengirola (n = 152) and Fuentenueva (n = 101) and any other locality.

## Discussion

This study is a field investigation aimed to unveil the role of major factors potentially affecting the captures of the invasive Asian tiger mosquito, *Ae. albopictus*, in an area of recent invasion in the provinces of Granada and Malaga (southern Spain)^23^. To that end, we regularly sampled *Ae. albopictus* and other autochthonous mosquito species between May and November 2022, at five different localities categorized as natural, urban, and peri-urban sites. These results provide valuable information for the monitoring and control of invasive mosquitoes with public health relevance.

### Effects of trap type and sex on mosquito captures

Our study emphasizes the higher efficacy of BG-Sentinel traps over CDC-UV traps in capturing mosquitoes in our study area. The BG-Sentinel traps effectively captured *Ae. albopictus* in nearly 50% of the trapping events, while CDC traps achieved a success rate of 5%. Although both traps had a higher overall efficiency in capturing *Cx. pipiens* than *Ae. albopictus*, significant differences between trap types persisted. Indeed, the higher effectiveness of BG-Sentinel traps compared to other trapping methods has been extensively demonstrated in various environments and for different mosquito species^26–30^. Certain widely used mosquito traps (e.g., CDC miniature light traps, gravid traps, or New Jersey light traps) have been found to be inefficient in capturing *Ae. albopictus*^26,27,29^. This may be because this invasive species is predominantly diurnal and seeks hosts near the ground surface^31,32^. Here, CDC-UV traps were placed at approximately 1.5 m above the ground (but see, for example^30,33^, for alternative settings), that may enhance their efficacy in capturing mosquitoes with more crepuscular or nocturnal activity patterns, such as *Cx. pipiens*^30^. Additionally, the use of visual and olfactory attractants, such as the BG-Lure and CO_2_, employed in BG-Sentinel traps, may further improve the capture of female mosquitoes of both species^26,29^. So, these results support the necessity to consider the capture method in order to compare mosquito captures between different studies. Interestingly, both types of traps demonstrated comparable performance in capturing *Cx. pipiens* males, whereas male *Ae. albopictus* were more commonly captured using BG-Sentinel traps. This difference can be attributed to the specific behavior of *Ae. albopictus* males, who, in order to increase their mating chances, adeptly differentiate the chemical and olfactory signals emitted by hosts, anticipating the presence of females^34^. Lastly, our findings support the general pattern previously reported in various mosquito species, where female mosquitoes outnumber males in captures^26–29,35^.

### Effects of locality and seasonality on mosquito BG-captures

The abundance of both *Ae. albopictus* and *Cx. pipiens* mosquitoes was determined by the sampling locality, with varying effects observed between sexes of the latter species (Fig. 2, Table 1). The Bioparc zoological garden, located in the urban area of Fuengirola (Malaga province), had the highest abundance of *Ae. albopictus*. Zoological gardens may provide suitable habitats for mosquitoes, especially for invasive species like *Ae. albopictus*. This is due to the availability of breeding sites (e.g., puddles on the ground or small water holes on plant surfaces, such as in the cut stems of lucky bamboo), the presence of exotic plants that mimic the southeast Asian landscapes where the species is native, and the abundance of potential hosts. These factors enable continuity and proliferation of the mosquito populations within the region^36,37^. *Aedes albopictus* is capable of blood-feeding on various organisms, including fish, reptiles, and birds. However, the majority of its blood meals come from mammals, with humans recognized as a common host of this species^38,39^. This fact supports the role of *Ae. albopictus* as an important human nuisance but also a potential vector playing a role in the transmission of pathogens, including those affecting wildlife and humans such as *Dirofilaria*^13,14^. Moreover, differences in the abundance of *Ae. albopictus* mosquitoes between Fuengirola (11 m.a.s.l.; Table 3), situated by the sea, and localities in Granada (ranging between 642 and 762 m.a.s.l.; Table 3), could be attributed to climatic differences. The former features milder environmental conditions in contrast to the more extreme and variable conditions present in the localities of Granada^31,40–42^. Considering that the period of mosquito captures in the zoological garden was shorter (traps operated less hours than in the other localities due to the presence of visitors; see methods), much higher differences could be expected if mosquitoes were captured over a 24-h period. Finally, we recorded the presence of *Ae. albopictus* in all localities of Granada, with significantly higher abundances in the peri-urban site of Gójar compared to the natural site of La Vega.

**Table 3:** Characteristics of the sampling localities included in this study in the provinces of Granada (four sampling localities) and Malaga (one locality).

Sampling locality	Province	Habitat type	Natural area (%)	Urban area (%)	(people/km^2^)	Coordinates		Altitude (m.a.s.l.)
						Latitude	Longitude	
Fuengirola	Malaga	Urban	0	100	15,045	36° 32′ 16.7″ N	4° 37′ 39.5″ W	11
Fuentenueva	Granada	Urban	0	100	7666	37° 10′ 50.4″ N	3° 36′ 32.7″ W	663
Cartuja	Granada	Periurban	21	79	587	37° 11′ 38.7″ N	3° 35′ 51.2″ W	757
Gójar	Granada	Periurban	55	45	924	37° 06′ 40.2″ N	3° 36′ 22.4″ W	762
La Vega	Granada	Natural	100	0	5	37° 09′ 57.6″ N	3° 37′ 27.6″ W	642

*m.a.s.l.*: meters above sea level.

A previous study developed in Andalusia (Spain) demonstrated that, although *Cx. pipiens* is the predominant mosquito species in urbanized areas, it also exhibits higher abundance in rural and natural areas^43^. We found significant differences in the abundance of female *Cx. pipiens* between the natural site of La Vega, the locality with the highest number of captures of this species, with respect to the peri-urbans sites of Cartuja and Gójar (Fig. 2, Table 1). La Vega, a waste-water treatment plant, is surrounded by agriculture fields providing suitable habitats for the breeding of this mosquito species. In urban sites such as Fuengirola and Fuentenueva, the abundance of *Cx. pipiens* was similar to that in peri-urban sites.

For males, the lowest abundance of *Cx. pipiens* was also detected in Cartuja, an open and highly exposed sampling point in the peri-urban area of Granada. The lack of correlation among localities in male and female *Cx. pipiens* abundances may be explained by the different ecological requirements and behavior of these sexes. Male *Cx. pipiens*, tending to remain near breeding sites, exhibit lower dispersal than females, which actively seek out hosts^44^. Nevertheless, the results obtained for *Cx. pipiens*, together with those found for *Ae. albopictus*, suggest that local-scale environmental characteristics, such as microhabitat characteristics suitable for mosquito breeding and temperature or rainfall^40–42,45,46^, may strongly determine the presence and abundance of these mosquito species. While *Cx. pipiens* is a common species in urban sites, it reaches its maximum abundance in natural habitats^43,45–47^, whereas *Ae. albopictus* shows a higher preference for urban sites with milder winters and water infrastructures^31,40–42^.

Finally, we did not find significant differences in the captures of *Ae. albopictus* mosquitoes between trapping sessions. However, the abundance of this species reached its maximum from mid-July to late September (sessions 4–7; Fig. 2), representing almost 75% (100/135) of females and 72% (46/64) of males captured. This seasonality is similar to those previously reported in field surveys in other areas of southern Europe^29,35,48^. For instance, a study conducted in 2019 within the Portuguese Algarve, geographically proximate to our study area, observed that the peak abundance of adult mosquitoes was reached between mid-July and mid-September^35^. In such study, two short peaks were detected at early October and early November 2019, which were not registered in our field samplings. The differences in experimental designs and the extreme weather conditions observed in October and November 2022 in the provinces of Malaga and Granada, where they were the driest and hottest on record, may account for these findings^49^. The relatively small number of *Ae. albopictus* mosquitoes caught during the study period, especially in the first and last sampling sessions, might account, to some extent, for the absence of variations in overall mosquito captures.

*Culex pipiens* seasonality has been extensively documented. We observed that population dynamics align with prior field surveys conducted in Spain and other countries within the Mediterranean basin^45–47^. Compared to *Ae. albopictus*, we identified a more consistent and stable seasonal pattern on *Cx. pipiens*, with more subtle variations along the seasons. Two peaks in abundance were identified: one in May through early June (sessions 1–2; Fig. 2) and a slightly larger peak from September through the end of November (sessions 7–10; Fig. 2), excluding the hottest summer months. Thus, although *Cx. pipiens* displays broad tolerance to environmental factors, our findings suggest that its populations may thrive in the wetter and warmer months, but face constraints under extreme heat conditions^45–47^.

The observed temporal dynamics for both species show some differences (Fig. 2), with their abundance peaks occurring at different times. These differences in temporal shifts may be attributed to niche differentiations related to climate and the availability of breeding sites in the area^50^. Yet, these results could be due to other factors such as interspecific competition between these species as *Ae. albopictus* may effectively compete against other species, including *Cx. pipiens*, during the larval stage^51^. A prior study of two mosquito species in natural environments of northern Italy provide support for the significant impact of interspecific competition and the temporal niche effect on the abundance patterns of both species^50^. This asymmetrical interspecific competition could lead to temporal changes in the dynamics of both species^50^. Nevertheless, complementary field surveys are necessary to identify the relative contributions of niche differentiation and interspecific competition to the temporal dynamics observed in our study.

In conclusion, *Ae. albopictus* is an important nuisance for human populations in the invaded area where it may also play a role as a potential vector of locally circulating and imported pathogens^40^. The species was introduced in southern Spain in recent decades and is currently experiencing a population increase. The native *Cx. pipiens* also represents a significant concern for wildlife and public health, as it is a proficient vector for pathogens such as the West Nile virus^47,52^. This virus has caused outbreaks in several Mediterranean countries in recent years, including the southern Iberian Peninsula. Our findings demonstrate that trap type, sex, and locality are significant factors that influence the captures of both invasive and native mosquito species. These findings have implications for monitoring and surveillance of local populations of the recently established *Ae. albopictus* and the autochthonous *Cx. pipiens* and, subsequently, to prevent their potential contribution to the transmission of locally circulating and imported pathogens in southern Spain.

## Methods

### Study area

Mosquito sampling was conducted from early May to late November 2022 in five locations throughout southern Spain, including one natural site, two peri-urban sites, and two urban sites (Table 3). One urban location, the Bioparc Zoological Garden, is located in the Malaga province, and the four additional sampling sites are in the Granada province. The study sites in Granada included a natural location near a sewage station surrounded by agricultural fields (the natural site of La Vega), an urban area situated on the Fuentenueva campus of the University of Granada (UGR), and two settings with an intermediate degree of urbanization: the periurban sites of Cartuja campus of the UGR and Gójar (Table 3; Fig. 2a). Localities were classified as urban, peri-urban, or natural sites based on population density and the percentage of natural/urban areas. In brief, land use and population density were obtained from http://www.juntadeandalucia.es/institutodeestadisticaycartografia/DERA/ and processed with QGIS v3.18.1^53^. First, we set up buffers of 500 m radius around each sampling point. To quantify the land use in each buffer, we used the 'disolve' and 'intersect' geoprocessing tools, and then the 'statistics by categories' tool, obtaining the total area and percentage of each variable per buffer. For the land use, we obtained a total of 8 categories for the 5 sampling points, which we grouped into 2 classes: natural areas, grouping the "*permanently irrigated land*", "*mainly agricultural land, but including natural vegetation*", "*olive groves*", "*crop mosaic*", and "*natural grasslands*" categories, and urban areas, grouping the "*industrial or commercial zones*", "*continuous urban fabric*", and "*discontinuous urban fabric*" categories. In the case of human population density, the data used was estimated as the number of people living in a grid of 250 × 250 m by the Institute of Statistics and Cartography of Andalusia from the latest local census from 2021. We used the 'intersect' geoprocessing tool to determine the percentage of each grid within each buffer and used the 'statistics by categories' to estimate the total population in these grids.

### Mosquito sampling

We conducted 10 sessions of mosquito capture in each of the five localities. At each sampling location and trapping session, two Blacklight (UV)-CDC Miniature traps (Centers for Disease Control and Prevention, Atlanta, USA) and two Biogents (BG)-sentinel-2 traps (Biogents, Regensburg, Germany) were set up. Consequently, each trap type was set 100 times throughout the sampling period. BG-Sentinel traps were supplemented with dry ice, as a source of CO_2_, and BG-Lure. Two sampling points were established at each locality, each with one of the two trap types. To minimize bias, sampling locations within localities were placed approximately 10–50 m apart, depending on the possibilities found in each locality. The selection criteria for these locations were determined by maintaining consistent environmental conditions. Ideally, traps were located in shaded and humid areas near water sources and vegetation and representative of the locality being sampled. Additionally, we assigned an individual number to each trap for individual identification and traps were alternated between the two sampling points within each locality to avoid potential bias associated to the trap identity. The sampling order of localities within each trapping session was randomized for the same purpose. Trapping sessions were conducted every 2–3 weeks, avoiding days with adverse conditions for mosquito sampling (e.g., rainy and windy days). Traps operated during 24 h in each trapping locality. However, due to the affluence of human visitors to the Fuengirola sampling locality (i.e., Bioparc zoological garden; Malaga province), in this site traps only operated from 19:00 pm to 10:00 am (local time).

Collected mosquitoes were transported to the laboratory on dry ice and maintained frozen (− 80 °C) until further analysis. Subsequently, mosquitoes were sexed and identified using morphological keys^54,55^.

### Statistical analyses

We focus this study on the invasive species *Aedes albopictus* and the common house mosquito *Culex pipiens*. Analyses were restricted to these species due to the limited number of captured mosquitoes from other species and in order to address the main focus of the study. Firstly, we performed a Pearson's chi-square test to evaluate the efficacy of BG-Sentinel and CDC-UV traps across the 100 deployments of each trap type. We compared the success of trapping events, that is, whether each mosquito species was captured or not (i.e., prevalence). Furthermore, as the data were not normally distributed, Mann–Whitney *U* tests were performed to compare the abundance of *Ae. albopictus* and *Cx. pipiens* mosquitoes captured among trap types. These analyses were restricted to mosquitoes identified to the species level and with their sex determined.

Due to the high amount of unsuccessful trapping events and the relatively low number of mosquitoes trapped using CDC-UV traps (see results and Table 1), only data corresponding to BG-Sentinel captures were included in the subsequent analyses. In this case, the total number of mosquitoes captured in the two BG-Sentinel traps per session was log + 1 transformed to meet the assumptions of GLM with Gaussian distribution^56^. Subsequently, we performed individual analyses for *Ae. albopictus* and *Cx. pipiens*, including abundance as the response variable and the categorical factors sex, locality, and trapping session, as well as their interactions, as independent variables. From these saturate models, we conducted a backward stepwise procedure to remove non-significant variables (*p* > 0.05). Thus, only significant variables remained in the final models. The contribution of each GLM term to the overall variance explained by the final models were calculated as eta-squared (η^2^). Tukey's post hoc tests were used to identify differences between levels of the categorical variables. Statistical analyses were run in R version 4.2.3^57^ using the ‘*lme4*′^58^ and ‘*emmeans*’^59^ packages.

### Ethics approval and consent to participate

Ethical approval was not required for this study according to national/local legislation because mosquitoes are not protected by any law. Written informed consent was obtained from the individual for the publication of any potentially identifiable images or data included in this article.

## Data Availability

The data that support the findings of this study are available from the corresponding authors, upon reasonable request.
