# Author Correction: *Aedes albopictus* in a recently invaded area in Spain: effects of trap type, locality, and season on mosquito captures

**DOI:** 10.1038/s41598-024-54211-9

**Published:** 2024-02-16

**Authors:** Mario Garrido, Jesús Veiga, Marta Garrigós, Manuel Morales‑Yuste, Jesús Recuero‑Gil, Josué Martínez‑de la Puente

**Affiliations:** 1 Department of Parasitology, University of Granada (UGR), Granada, Spain; 2 Bioparc Fuengirola, Fuengirola, Málaga Spain; 3 Doñana Biological Station (EBD, CSIC), Sevilla, Spain; 4 CIBER de Epidemiología y Salud Pública (CIBERESP), Madrid, Spain

Correction to: *Scientific Reports* 10.1038/s41598-024-52040-4, published online 25 January 2024

The Acknowledgements section in the original version of this Article was incomplete.

“This study was financed by the PID2020-118205GB-I00 grant to JMP funded by MCIN/AEI/10.13039/501100011033. Additional support derived from the CNS2022-135993 grant of the Ministerio de Ciencia e Innovación (MCIN/AEI/10.13039/501100011033) with funding from European Union NextGenerationEU. Mario Garrido was supported by the María Zambrano program and the P9 program for the Incorporation of Young Doctors funded by Spanish Ministry of Universities, the European Union-NextGenerationEU, and the University of Granada. Jesús Veiga received financial support from the Margarita Salas and Juan de la Cierva (FJC2021-048057-I) programs funded by Spanish Ministry of Universities, the Spanish Ministry of Science and Innovation and the European Union-NextGenerationEU. Marta Garrigós was supported by a FPI grant (PRE2021-098544). Mario Garrido is currently granted by the PID2022-137746NA-I00 funded by Spanish Ministry of Science and Innovation. We greatly appreciate the support given by the “Grupo de Investigación Comportamiento y Ecología Animal” of the University of Granada for the field sampling.”

now reads:

“This study was financed by the PID2020-118205GB-I00 grant to JMP funded by MCIN/AEI/10.13039/501100011033. Additional support derived from the CNS2022-135993 grant of the Ministerio de Ciencia e Innovación (MCIN/AEI/10.13039/501100011033) with funding from European Union NextGenerationEU. Mario Garrido was supported by the María Zambrano program and the P9 program for the Incorporation of Young Doctors funded by Spanish Ministry of Universities, the European Union-NextGenerationEU, and the University of Granada. Jesús Veiga received financial support from the Margarita Salas and Juan de la Cierva (FJC2021-048057-I) programs funded by Spanish Ministry of Universities, the Spanish Ministry of Science and Innovation and the European Union-NextGenerationEU. Marta Garrigós was supported by a FPI grant (PRE2021-098544). Mario Garrido is currently granted by the PID2022-137746NA-I00 funded by Spanish Ministry of Science and Innovation. We greatly appreciate the support given by the “Grupo de Investigación Comportamiento y Ecología Animal” of the University of Granada for the field sampling. This paper had been financed by Programa QUALIFICA, Junta de Andalucía, Consejería de Universidad, Investigación e Innovación (project QUAL21 020 EBD)"

The original Article has been corrected.

